# Women's mental health and COVID-19: increased vulnerability and inequalities

**DOI:** 10.3389/fgwh.2024.1414355

**Published:** 2024-10-02

**Authors:** Margareth Arilha, Adriana P. Carvalho, Thais A. Forster, Carla V. M. Rodrigues, Bianca Briguglio, Suzanne J. Serruya

**Affiliations:** ^1^ Elza Berquó Center for Population Studies, State University of Campinas (UNICAMP), Campinas, Brazil; ^2^ Department of Sociology, University of São Paulo (USP), São Paulo, Brazil; ^3^ Latin American Center for Perinatology, Women and Reproductive Health, Pan American Health Organization (PAHO), Montevideo, Uruguay; ^4^ Department of Sectoral Development, Brazilian Regulatory Agency for Private Plans (ANS), Rio de Janeiro, Brazil; ^5^ Labor Movens - Working conditions in Tourism, University of Brasília, Brasília, Brazil

**Keywords:** mental health, COVID-19, women, review, pandemics

## Abstract

**Introduction:**

The impact of COVID-19 on mental health has become a relevant object of research. Studies have demonstrated that women have experienced greater mental health challenges, highlighting the importance of public health systems to address women's specific needs.

**Methods:**

This literature review explores the effects of the coronavirus pandemic on psychological distress among women, aiming to provide a comprehensive understanding of the subject and to explore how these research findings can guide public mental health care responses in crisis settings. A total of 131 studies were analyzed and four dimensions were discussed: study characteristics, factors impacting women's mental health in the pandemic setting, particularities of pregnancy and the postpartum period, and proposed interventions. Most studies exclusively addressed populations of adult women, predominantly during pregnancy and the postpartum period.

**Results:**

Anxiety, depression, and stress were the most common outcomes. Lower education and income, preexisting mental health problems, and living alone or with children were risk factors for higher levels of anxiety and depression.

**Discussion:**

A comprehensive care approach supported by public health policies and focused on intersectional factors, including race, socioeconomic status, and access to resources, is necessary to improve women's mental health care response in future crises.

## Introduction

During the COVID-19 pandemic, the necessary measures of movement restriction, social isolation, and prioritization of treatment for patients with COVID-19 by health care services have profoundly changed individual and family routines and have aggravated the problem of inequality between population groups. Women are especially affected by these changes. Not only have they maintained their usual tasks, but have also accumulated activities involving education of their children and caring for older and/or sick family members. In many cases, they have lost or abandoned their jobs to meet the needs of their families.

Triple duty, children, work, shortage of material resources, lockdown and fear of illness, and, in some cases, violence have considerably increased the emotional burden women have had to deal with in their different ways of coping with the crisis. Additionally, they had no access to recreational and social activities or to health care services for matters of sexuality and reproductive health, pregnancy, delivery, and the postpartum period. These circumstances also seem to have deteriorated the preexisting state of their subjectivities, their position in the world, their perspectives on life, and their personal resources to face the new demands.

A systematic review titled “Global prevalence and burden of depressive and anxiety disorders in 204 countries and territories in 2020 due to the COVID-19 pandemic” (1) and published by The Lancet in October 2021 indicates that approximately 53 million cases of depression and 76 million cases of anxiety were due to the COVID-19 pandemic. The study also highlighted that women and young people were the most affected population groups, but that additional studies are required to determine the severity and duration of the impact. However, even before the pandemic, the leading causes of disease burden were anxiety and depressive disorders, as the study emphasized. A key methodological issue raised by the authors is the quality of the instruments available for such measurements, which makes the question of how to promote mental well-being and target mental health determinants even more complex. Similarly, “Strengthening mental health responses to COVID-19 in the Americas: A health policy analysis and recommendations,” (2) a study published in November 2021, reports that women and young people were substantially affected during the pandemic and that mental health should be consistently addressed by public health systems given the severity of the emotional burden people have experienced.

This is the gap that this study aims to fill: making the issue of women's mental health in the critical period of the pandemic more visible. Contrary to initial assumptions, the effects of the COVID-19 pandemic on different population groups were not homogeneous in view of social determinants hierarchically determined by gender, race/ethnicity, age, sexual orientation, and economic status structures. An estimated 72% of health workers who have lost their lives to the COVID-19 pandemic are women (3). Studies have shown that female health workers usually present with more symptoms of anxiety and depression, insomnia, and burnout given their working conditions, especially frontline female workers who must permanently wear personal protective equipment (4).

The Region of the Americas has surpassed 100 million cases of COVID-19 and accounts for most of the reported deaths. The catastrophic situation is associated with social, economic, and health issues (3, 5–7). Pregnant women are more vulnerable and tend to develop more severe symptoms. Approximately 20 million women in the Americas have stopped using contraceptives either because they cannot pay for them or because their sexual practices have changed during isolation, as physical encounters have become rarer, with possible psychological impacts (8).

The Pan American Health Organization (PAHO) stated in 2021 that continued disruption of women's health services due to COVID-19 could erase more than 20 years of progress in reducing maternal mortality and increasing access to contraceptive use. A Latin American Center for Perinatology/Women's Health and Reproductive Health study showed that maternal mortality increased during the pandemic, which slowed down the Region's achievements in the past decade (3).

According to the COVID-19 Sex-Disaggregated Data Tracker, the gender/sex system has determined important variations in the facts and consequences associated with the pandemic (9). It is worth mentioning a Brazilian article on maternal mortality from COVID-19 published in The Lancet Regional Health—Americas (10). The study revealed that failures in medical care for pregnant women with COVID-19 are exacerbated by racial and gender discrimination, especially with regard to sexual and reproductive health. The authors found, for example, that interventions for preserving women's lives, such as intubation and preterm labor induction, were postponed by physicians for awaiting fetal development.

Importantly, the course of the first period of the pandemic has showed that the configurations of social, political, cultural, and economic practices that determine how people fall ill and die also pave the way for a greater chance of survival for women and men during the COVID-19 pandemic. There have been many impacts in terms of access to health care services and vaccinations, revealing relevant gender variations in pandemic management results.

In this context, the present study aimed to map the discussion about the influence of COVID-19 on women's mental health based on the literature published in the first period of the pandemic and to identify how scientific research was produced with a description of the main results.

### Women, mental disorders, and subjectivities: a conceptual tour

In recent years, the United Nations has made important efforts in an attempt to understand the issue. A PAHO study titled “The Burden of Mental Disorders in the Region of the Americas, 2018” presented an overview of perceived disabilities from substance use and specific neurological disorders and self-harm, combined or not with premature mortality, the imbalance between mental health spending and the related disease burden, and the limited allocation of mental health resources by countries of the Region (11). The study revealed that depressive disorders account for 7.8% of total years lived with disability (YLDs), while self-harm and pain disorders account for 4.7% of YLDs. The paper is also part of a process that, albeit slow, has managed to add the concern with the burden of mental distress to the global agenda, with the introduction of specific mental health indicators in the Sustainable Development Goals (SDGs) and the 2030 Agenda (12). SDG 3, which refers to health issues, specifically established an indicator for reducing suicide mortality rates by 2030. Additionally, there is a specific goal for strengthening the prevention and treatment of substance abuse, including narcotic drug abuse and harmful use of alcohol.

Although the burden of anxiety, depression, and self-harm among women has grown significantly in recent decades, few studies have focused on analyzing the evidence systematically and consistently. Of all WHO mental health publications (13–29), only two (17, 21) refer specifically to the female population. The Lancet Psychiatry Series on Women's Mental Health aims to explore social and biological factors in an attempt to understand the so-called mental disorders in women (30). As the Series describes, although some elements of the prevalence and pattern of psychological problems in women are known, the underlying reasons are still under-researched. Thus, a certain group of psychoses, depressive disorders, including perinatal and postpartum depression, anxiety disorders, and issues associated with gender violence are addressed, but in a very controversial way if a gender lens is used to interpret the findings.

Women are at the center of all new and complex situations involving panic, depression, anxiety, bereavement, and sadness. Concomitantly, they seek to preserve their life projects, their affective-sexual bonds, their personal perspectives on productive and reproductive life, and especially their attitude towards facing isolation and losses in the family, community, and relationship contexts. This emotional turbulence accumulates in their daily lives, often with no space to be expressed. Countless were the demands of life adaptation for finding new possibilities of survival.

To meet the challenge of addressing human suffering in this context, we must review the notion of subject that each disciplinary field adopts. This is a process of conceptual review that makes it possible to overcome a fragmented view of each of the subjects in question and approach them — in this case, women — in their singularities, multidetermined by social factors and inserted into their configuration of language, their own network of meanings and signifiers. Such perspective could remove women from the place of carriers of disorders and make them subjects fully capable of operating with their own emotional life in processes of listening.

Many authors have proposed a shift from the idea of a subject defined by disorders to that of a subject defined by desires and rights. According to Birman, distress and trauma translate into helplessness and dismay in the subjects (women) who need spaces to manage their own psyche and favor the crucial transformation of pain into words (language) (31). The role of public policies is to bring to the forefront, as Birman indicates, “the possibility for the subject, as a citizen, to be able to count on the Other, to trust the ruler as an unmistakable real and psychic instance of protection in the face of a misfortune for defending life and thus preventing the possible advent of death” (free translation). Thus, distress as experienced by the citizens would be appeased by means of concrete actions. If the pandemic has exacerbated a group of psychological and psychiatric conditions such as panic syndromes, obsessions, phobias, and eating disorders, among others, what specific meanings have been triggered in the singular stories of each woman in their coping, distancing, and caring processes in the pandemic? What is the place of anxiety and depression, two famous conditions since the second half of the past century, in this context (32, 33)?

Intertwining these issues with those referring to sociocultural structures that codetermine cultural foundations, ie, the field of inequalities, is a key feature of our perspective. Working with social determinants is a strategy that has been developed by the WHO since 2005, when the Commission on Social Determinants of Health was created. In this context, several sociocultural determinants cooperate to create health inequalities and, in our view, should be considered markers of the language of subjects who seek acceptance in any clinical setting. However, it is not only a matter of accepting that subjects are constructed in specific hierarchical cultures with distinct established power relations, but that the different determinants themselves construct the body of identifications that each one carries inside and ultimately operate in the formation of subjectivities, of the singularity of each subject. This comprehensive perspective of psychological distress leads to some questions, including, how public health emergencies, as the COVID-19 pandemic, impacts women's mental health?

## Methods

A literature review based on the scoping review method, a strategy employed to map the extent and nature of publications in a given research area, was conducted (34, 35). The PubMed and Scopus databases were searched using the terms “COVID-19,” “SARS-CoV-2,” “Woman,” “Women,” and variations of the term “mental health” combined with specific terms related to subjectivity, violence, and sexual and reproductive health. Articles published between January 1, 2020 and June 30, 2021 in English, Portuguese, and Spanish with any type of study design were included; the bibliographic search was carried out in August 2021 (Supplementary Material S1 and S2).

Publications were considered eligible for inclusion if they reported at least one mental health outcome and mentioned specific data on women. For the purposes of this study, mental health outcome is understood as the group of most common symptoms in the psychiatric literature and in the fields of clinical psychology, psychiatry, and psychoanalysis, extending to a set of terms and concepts commonly used in these fields, such as anguish, melancholy, bereavement, and psychological distress, among others.

General population studies not reporting specific results on women, studies focusing on specific conditions (cancer, hypertension, diabetes, rheumatism, and others), and studies addressing specific professional categories, except health workers in public health fields, teachers, and workers linked to a single institution, were excluded. The resulting publications were submitted to full-text screening, and final selection was determined by agreement of at least two team members. Disagreements were resolved by a researcher with a background in women's mental health. The inclusion and exclusion criteria are detailed in Supplementary Material S3.

**Figure 1:**
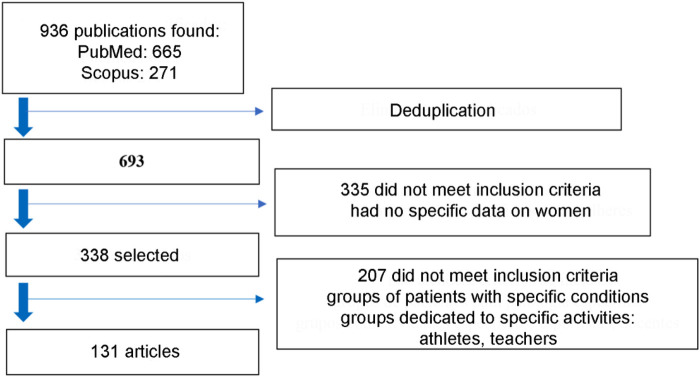
Flow diagram of article selection.

Initially, 936 publications were found; after deduplication and screening, 131 publications were included in the review (Figure 1).

The selected studies were organized with the aid of Mendeley, and the data were extracted by three researchers and later revised for greater precision. The following data were extracted: basic information (authorship, country and date, year of publication); study design (methods, inclusion criteria, exclusion criteria, sample, baseline sample characteristics, and analyzed outcomes); main results; risk and protective factors associated with mental health problems; recommendations; limitations; and funding. The characteristics of the publications are described in Supplementary Material S3, together with mental health outcomes and main results of interest for this review.

The content extracted from the publications was explored through quantification, categorization, and comparison strategies. Four dimensions are highlighted in the results, namely study characteristics, factors impacting women's mental health in the pandemic setting, particularities of pregnancy and the postpartum period, and proposed interventions.

## Results

### Study characteristics

Of 131 publications selected, 58 refer to the first year of the pandemic (2020) and 73 to the first half of 2021, ending on June 30, 2021. Europe (*n* = 41), North America (*n* = 29), Asia (*n* = 22), and the Middle East (*n* = 21) account for most of the publications. South America accounts for 12 studies, with Brazil being the leading country in number of publications. Africa and Oceania were the bottom regions, ranging from one to four publications. Two multicountry studies, one including 49 countries (36) and the other including four countries (Ethiopia, India, Peru, and Vietnam) (37), were selected. Overall, the United States and China were the leading countries, with 17 and 16 studies, respectively. Study characteristics are summarized in Table 1.

**Table 1:** Summary of study characteristics.

Characteristic	Studies (n)	Studies (references)
Year of publication		
2020	58	(38–95)
2021	73	(36, 37, 96–166)
Study region		
North America – Canada, United States, and Mexico	29	(37, 39, 50, 54, 55, 62, 72, 81, 82, 91, 104, 107, 114–116, 125, 132, 134, 135, 142, 146, 148, 150–152, 155, 157, 158, 165)
Europe – Belgium, Spain, Finland, France, Netherlands, Hungary, England, United Kingdom, Ireland, Italy, Norway, Poland, Portugal, and Sweden	41s	(40, 42–45, 47, 48, 51, 57, 59, 60, 65, 66, 69, 73, 75, 77, 79, 83, 86, 87, 90, 98–100, 102, 103, 108, 111, 113, 122, 123, 127, 128, 130, 131, 136, 146, 154, 160, 166)
Asia – Bangladesh, China, India, Vietnam, Japan, and Singapore	22	(49, 52, 53, 56, 58, 61, 63, 92–97, 106, 112, 124, 129, 149, 156, 159, 161, 162)
Middle East – Iran, Cyprus, Israel, Lebanon, and Turkey	21	(38, 67, 68, 74, 78, 84, 88, 89, 101, 105, 117–121, 137, 138, 140, 144, 163, 164)
South America – Argentina, Brazil, Colombia, Ecuador, and Peru	12	(36, 46, 70, 71, 85, 109, 110, 129, 133, 143, 145, 147)
Oceania – Australia and New Zealand	4	(41, 64, 126, 139)
Africa – Ethiopia and Tunisia	2	(80, 129)
Study design		
Cross-sectional	85	(36–38, 40, 42, 43, 45–48, 50, 52–57, 59, 62, 63, 65–67, 69–75, 78, 80, 83, 86–89, 92, 93, 95–97, 99–101, 103–108, 110, 112–114, 117, 120, 121, 124, 126, 128, 130, 133, 136–140, 142–144, 147, 148, 151, 152, 154–158, 161–164, 166)
Longitudinal	22	(39, 49, 60, 68, 79, 81, 82, 84, 98, 102, 109, 111, 116, 122, 123, 129, 131, 132, 141, 159, 160, 165)
Randomized controlled trial	1	(146)
Systematic literature review	7	(58, 64, 77, 94, 145, 149, 153)
Nonsystematic literature review	1	(91)
Exploratory	2	(51, 127)
Descriptive phenomenological	1	(119)
Digital ethnography	2	(41, 150)
Reflective analysis	1	(85)
Mixed methods	1	(44)
Commentary	3	(76, 125, 135)
Letter	1	(61)
News feature	1	(90)
Qualitative not specified	2	(118, 134)
Quantitative not specified	1	(115)
Participants		
Pregnant and postpartum women	57	(39, 41, 54, 56, 60–63, 66, 68, 70, 72, 73, 77, 81, 82, 85, 86, 88, 91, 93–95, 105, 107, 109, 110, 113–115, 117–123, 125, 132, 134–137, 139, 140, 146, 148–150, 152, 153, 155, 156, 159, 162, 164, 166)
Women undergoing fertility treatment	4	(38, 50, 104, 142)
Adult women	7	(44, 74, 76, 79, 80, 90, 127)
General adult population	42	(36, 40, 46–49, 51, 59, 65, 67, 69, 75, 78, 83, 84, 87, 92, 97–99, 101–103, 106, 108, 111, 112, 128, 130, 131, 133, 141, 143, 144, 147, 151, 157, 158, 160, 161, 163, 165)
Health professionals	10	(37, 42, 52, 53, 57, 58, 96, 116, 145, 154)
Adults with a psychiatric diagnosis	2	(55, 89)
University students	2	(43, 124)
Young people	2	(126, 129)
Adults with confirmed or suspected COVID-19	2	(71, 138)
Adults with HIV/AIDS	1	(100)
Adults working at home	1	(64)
Couples undergoing infertility treatment	1	(45)

A wide variety of sample sizes was observed; in studies on pregnant women, the sample ranged from 15 (118) to 15,428 individuals (156), while in studies including health professionals, the number ranged from 204 (116) to 12,596 individuals (96). The sample population was heterogeneous in more than 80% of studies.

When geographic distribution was analyzed by year of publication, China, Spain, and Italy were the countries that produced most research (36%) on COVID-19 and women's mental health during the first ten months of the pandemic, taking March 2020 as the beginning of the pandemic. These countries were greatly affected in the first period of the pandemic. It is worth noting that most empirical studies were conducted in the first half of 2020 (*n* = 106), which requires caution in using the results for later stages of the pandemic. Only a few Chinese studies (58, 149, 159) were initiated as early as 2019, considering that the virus was first detected in the Chinese province of Wuhan. Even among those published in 2021, most studies were conducted in the first months of 2020.

The group of analyzed publications consists mostly of empirical studies (*n* = 119), with a predominance of cross-sectional (*n* = 85) and longitudinal (*n* = 23) designs. Of the remaining studies, seven systematically reviewed previously published articles on mental health issues and the COVID-19 pandemic (58, 64, 77, 94, 145, 149, 153). The other publications covered the modalities of nonsystematic literature review, letter, commentary, and report. With regard to methods, the vast majority used online or telephone surveys/questionnaires to produce data, and only ten studies used some qualitative methodological strategy. Only one American randomized controlled trial was included, and the study involved 101 pregnant women, 50 in the intervention group (Calm meditation application) and 51 in the control group (146).

Qualitative investigations were dedicated to understanding how women experienced the pandemic, particularly focusing on social and family relationships (127), emotional and psychological consequences (51, 142), and specific experiences related to pregnancy and the postpartum period (41, 118, 119, 134, 139, 150). Difficulties in accessing abortion services during the pandemic were the object of mixed methods studies (44). In these studies, women's perceptions and experiences were captured through semi structured or in-depth interviews conducted by telephone or mobile instant messaging application (WhatsApp) and open online questionnaires, in addition to digital ethnography.

Most publications (52%) exclusively addressed adult women, predominantly pregnant and postpartum women (*n* = 57 or 43%). Studies on specific groups of women included those undergoing infertility treatment (38, 50, 104, 142) and married women with children (74).

Studies on the general adult population (31%) included health professionals, university students, people with mental health problems, people with COVID-19, people working from home, people with HIV/AIDS, and couples undergoing infertility treatment. Although the adolescent population was included in the search strategy used to find relevant articles, no publication analyzed in the screening stage reported specific results for female adolescents. Thus, no publications specifically addressing this audience were included in the review.

Of the topics that seemed to be of greatest interest, anxiety (*n* = 62), depression (*n* = 59), and stress (*n* = 32) were the most common outcomes, with many studies assessing more than one mental health outcome. Most studies used online surveys containing items from validated scales, which classify the severity of mental disorders according to different predefined levels, such as “mild,” “moderate,” and “severe.”

### Factors impacting women's mental health

Most studies reported that women and young people are the groups most affected by mental distress during the pandemic. As an exception, a Chinese study observed higher levels of risk of mental disorder in men than in women (92).

Several factors seem to be key to women's mental health status in the pandemic context (Supplementary Material S4), and they can be grouped into five dimensions: sociodemographic (age, education, region of residence), economic (work, income), health aspects (history of mental health and chronic diseases, physical activities, psychological treatment, pregnancy and the postpartum period); social aspects (marital status, family composition, social relationships during the pandemic); and pandemic-specific factors (concern with being infected, job loss, poor housing conditions to face isolation — the latter was much debated, especially at the beginning of the pandemic).

Lower education and income, preexisting mental health problems, and living alone or with children were all risk factors for higher levels of anxiety and depression at the beginning of social restrictions. In some countries, such as England, many of these inequalities were reduced as lockdown continued, but differences were still evident 20 weeks after social isolation began (102). Pandemic-specific factors (social isolation, financial insecurity, job loss, and lack of access to basic goods and services) were associated with virtually all mental health outcomes assessed in the studies.

One study reported that women with lower household incomes had consistently worse mental health than those with higher household incomes (102), which seems to be related to a higher number of adverse experiences, such as job losses, income cuts, and inability to pay bills. Such changes were primarily driven by the financial impact of the pandemic and the challenges of balancing child schoolwork and working from home and lack of childcare (132).

However, there is evidence that people with a higher socioeconomic status may experience a greater increase in depressive symptoms and a decrease in life satisfaction during the COVID-19 pandemic compared with those with a lower socioeconomic status (167).

Another study showed that women are more anxious about the financial situation of close relatives than about themselves and that health-related anxiety was greater than economic-related anxiety (59). The study also found that men and women did not differ significantly in terms of economic-related anxiety. This result particularly contrasts with the findings of other studies, such as a United Kingdom study including a larger sample that observed considerably higher levels of distress in women experiencing economic insecurity (83). A single study showed differences in this regard. Liu et al. revealed that in China, because of cultural determinants, young married men had more psychological symptoms during the pandemic than young women (106). Such distress is associated with financial concerns, and this apparently is an important gender difference in that country.

Regarding the maintenance of social and family relationships, a study on Italian women reported that, during social isolation imposed by the pandemic, women perceived the home as a safe place despite an increase in family conflicts. This shows that family relationships are resilient and, even at difficult times, allow members to overcome loneliness, keep company, and take care of each other (127).

A study assessing the influence of gender differences on housework and psychological distress in the United Kingdom revealed that women spent much more time on unpaid care work than men during lockdown. Also, it was more likely to be the mother than the father who reduced working hours or changed employment schedules because of increased time spent on childcare (160). Women who spent long hours on housework and childcare were more likely to report increased levels of psychological distress. Working parents who adapted their work patterns showed increased psychological distress compared to those who did not. This association was much stronger if he or she was the only family member who adapted work patterns or if she was a single mother. Fathers who reduced working hours showed increased psychological distress if mothers did not. There are gender inequalities in the divisions of unpaid labor. Balancing working from home with remote schooling and childcare, as well as extra housework, is likely to pose health problems for people with families, especially for mothers.

### Pregnancy and the postpartum period

Considering an extract of 57 articles whose population was pregnant women, including the perinatal and postpartum periods, approximately 30 different outcomes were assessed by either validated questionnaires or questionnaires developed by the authors. The most common outcomes in this extract (*n* = 62) included anxiety (*n* = 26), depression (*n* = 25), stress (*n* = 11), and postpartum stress and post-traumatic stress disorder (PTSD) (*n* = 11). Of note, studies may assess more than one outcome (Table 2). Less frequently assessed outcomes consisted of quality of life, well-being, sleep disorders, domestic violence, sexual function, psychological distress, pregnancy distress, satisfaction with childbirth, fear of childbirth, physical activity, and postpartum mood, among others.

**Table 2:** Most common outcomes in the extract of articles addressing pregnancy and the postpartum period (*n* = 62).

Evaluated outcome	*n*	%
Anxiety	26	42%
Depression and postpartum depression	25	40%
Stress, postpartum stress, and PTSD^a^	11	18%

^a^ Post-traumatic stress disorder.

In total, 43 research instruments were used to measure the outcomes listed in the extract of 57 articles, with the Edinburgh Postnatal Depression Scale (EPDS) (*n* = 12) being the most frequently used in the studies (*n* = 35). Other instruments are described in Table 3.

**Table 3:** Most common research instruments in the extract of articles addressing pregnancy and the postpartum period (*n* = 35).

Research instrument	*n*	%
Edinburgh Postnatal Depression Scale (EPDS)	12	34.3%
State-Trait Anxiety Inventory (STAI)	5	14.3%
Perceived Stress Scale (PSS-10)	4	11.5%
12-Item Short Form Health Survey (SF-12)	2	5.7%
Female Sexual Function Index (FSFI)	2	5.7%
Impact of Event Scale - Revised (IES-R)	2	5.7%
Hospital Anxiety and Depression Scale (HADS)	2	5.7%
Pandemic-Related Pregnancy Stress Scale (PREPS)	2	5.7%
Patient Health Questionnaire (PHQ-9)	2	5.7%
General Anxiety Disorder scale (GAD-7)	2	5.7%

With regard to prevalence, the level of psychological distress observed in pregnant and postpartum women exceeded that normally expected during pregnancy and that experienced by other groups during the current pandemic (39, 54, 56, 60). In contrast, one study found no difference in the levels of anxiety and depression in pregnant women before and after the onset of COVID-19 (166), and another found more symptoms of depression in nonpregnant women (164).

In pregnant women, specific concerns about COVID-19 affecting fetal health and prenatal care are associated with psychological distress, as well as concerns about changes imposed by the pandemic in childbirth conditions, especially with regard to the presence of a companion in the hospital. As noted, pregnant women are provided with inadequate and insufficient information about the effects of COVID-19 on pregnancy (63), which accentuates feelings of insecurity. In addition to fear and uncertainty about the clinical effects of COVID-19, these women often feel isolated, prevented from being with family or friends at a time when they long for more support (135). Interestingly, few authors (88, 135) addressed increased pressure on more vulnerable women, such as black and cultural minority women, who are already fighting social and health inequalities.

A lower risk of postpartum depression was observed among women who gave birth during strict isolation compared to those who gave birth before the pandemic (68). Probably because the study was conducted during isolation, mothers obtained greater support from family members who did not go to work or worked from home, and this gave them more opportunities to support pregnant women. Conversely, a Canadian longitudinal study demonstrated an increase in maternal depressive and anxiety symptoms in the first months of the COVID-19 pandemic (May to July 2020) compared to prepandemic periods (previous 10 years) (132). The findings suggest that this increase was universal, regardless of women's previous mental health history. These changes were primarily driven by the financial impact of the pandemic and challenges balancing child schoolwork and working from home and lack of childcare.

### Proposed interventions

The use of technologies (mobile applications, remote consultations via telephone or video, etc.) to offer support to the most vulnerable groups, especially pregnant women, postpartum women, and people with a history of mental health problems, is regarded as a fundamental strategy for identifying risk situations and for providing care services. Specifically, studies suggested that alternative interaction strategies be used to offer counseling (119, 157, 158, 162), telepsychiatry (78), online psychotherapy (75, 128, 147), support groups, and online education activities focusing on developing skills that favor problem-solving and stress management (75, 128, 136, 143, 158, 162). Interventions need to be adjusted over time to suit the different stages of the pandemic (75).

Most studies on pregnant and postpartum women showed that screening and treatment actions for depression and anxiety should be prioritized in this group. Another relevant point is the importance of creating spaces where pregnant women can receive quality information about the risks of COVID-19 during pregnancy and the postpartum period, address the fear of childbirth, and exchange concerns with other women (41, 70, 85, 156). It is highlighted that pregnant women who remain in isolation should be able to maintain regular communication with their loved ones through social media and receive adequate social support. More intensive therapies (such as cognitive behavioral therapy and adaptive skills training) may be necessary for those experiencing severe anxiety or depressive symptoms. Specific educational strategies addressing COVID-19 and its effects on pregnancy are needed for young pregnant women with less education and public health insurance to help maintain their psychological well-being (70).

Studies also emphasize that public health departments should provide a central information repository that is accessible, reliable, and welcoming to the population (46, 157). These strategies may involve expanding telephone helplines, sending mobile messages with qualified and non-stigmatizing information about COVID-19, providing information on protective measures, providing updates and real-time reports on the pandemic, and disseminating information on how to access urgent medical services and alternative means of interpersonal communication (48, 71, 127, 129, 150, 161, 163). However, it is necessary to guide the population on how to deal with anxiety caused by information overload (65, 75).

Employers also play an important role in the creation of support networks within organizations with the purpose of favoring an inclusive and supportive organizational culture (157). Specifically, companies should provide psychological and childcare support to female workers during the pandemic. Thus, employers are recommended to sponsor childcare programs, including collaborative home-based childcare services, to address school closures (37, 160). Strategies also need to ensure that people who choose or are forced to work from home do not experience negative career consequences, such as not being offered job promotions or training opportunities (64). At the same time, government institutions should ensure financial security, expand job opportunities, and provide public childcare services to support maternal mental health and child well-being (81, 83, 132, 157).

Governments are encouraged to include essential services to address violence against women in COVID-19 response plans, identifying strategies to make them accessible despite social distancing (76). Also, basic material and financial benefits should be provided by governments to guarantee people's quality of life and enable the adoption of preventive measures. Expanding income transfer programs and waiving conditionalities to increase access for vulnerable groups are highlighted strategies (71, 83, 92, 129). Creating community mental health programs for future outbreaks of infectious diseases is another relevant strategy (144).

In the research field, COVID-19 scientific councils, available in many countries, should be strengthened with scientists from different backgrounds, such as psychology, psychiatry, sociology, pedagogy, and education, for a broader and more adequate assessment of the impact of taken measures (163).

## Discussion

The multiple consequences of the COVID-19 pandemic on women's daily lives have generated expectations about the production of knowledge on how this context has impacted mental health in the female population. There has been a profusion of studies, including those that are analyzed here, showing that women have experienced significant psychological distress during the course of the pandemic, which applies to those who already had a history of mental disorders and those who developed psychological symptoms from the pandemic.

Most studies demonstrated that women and young people were the groups most affected by mental distress in the pandemic, confirming the results of other investigations that reported substantially worsening levels of psychological distress in these population groups in the pandemic setting (1, 98).

The characterization of risk factors that psychologically affect women is consistent with those of previous studies conducted before the onset of COVID-19 and outside the context of global health crises, which examined the impact of sources of psychosocial and socioeconomic stress on mental health (168). The so-called risk factors can be considered aspects of inequalities through which women's distress could be discursively circumscribed, and they include low education, low income, lack of support, no partnerships, difficulties in reconciling work and childcare, and lack of access to public services, among others.

The social isolation disproportionately affected women, forcing them into prolonged coexistence with family members who may not have had friendly or cordial relationships. The constant psychological strain, exacerbated by the fear of illness and death, led to numerous conflicts within families and communities. Women, regardless of their role as heads of household, were burdened with caregiving responsibilities, often going to great lengths to protect their families from contamination. The risk of illness or death, often more acutely understood by women themselves, fueled tensions within families. The pressure to maintain protective and caring functions for adults, especially the elderly, children, and adolescents, during these extraordinarily demanding times, further increased women's psychological burden. Women who were able to maintain their jobs faced the added strain of providing financially for their families. Moreover, the isolation from friends, family, and community support networks exacerbated the challenges faced by women.

The approach to domestic violence and its impacts in the pandemic context, or to self-inflicted violence — for example, a study on Japanese university students showing the burden on young women — was very limited in the group of studies, despite the relevance of these topics in women's lives. Reports indicate that social isolation and confinement measures during the pandemic led to increased incidents of domestic violence, contributing to psychological distress among women (169–171). The psychological impact of violence, coupled with limited access to healthcare services and support systems, has exacerbated womeńs mental health struggles.

It is noteworthy that the findings regarding the impact of economic factors on mental health are contradictory. A significant body of literature supports the notion that lower socioeconomic status is associated with poorer mental health outcomes, a conclusion supported by some studies examined. However, evidence was found suggesting that higher socioeconomic status may be linked to greater increase in depressive symptoms during the pandemic, when compared to lower socioeconomic status. This contradiction can be better understood by considering variations in the underlying causes of mental health issues during the pandemic. Women with lower socioeconomic status were generally associated with poorer mental health outcomes due to the financial impact of the pandemic and the challenges of balancing child schoolwork, working from home and lack of childcare. On the other hand, upper socioeconomic class was linked to mental health challenges possibly due to higher pressure to maintain status and greater disruptions in lifestyle. It's important to point out that contradictions in the findings could also be attributed to methodological differences between studies (sample populations, measurement tools and the mental health outcomes assessed), as well as to contextual factors, such as cultural differences.

Particularly regarding economic-related anxiety, the results show contrasting patterns between gender in different cultural settings. For example, a United Kingdom study observed higher levels of distress among women (83), while in China young married men had more psychological symptoms during the pandemic than young women (106).

Another relevant gender disparity lies in the domain of work. Women allocated significantly more time to unpaid care work than men during lockdowns, leading to greater psychological distress. The challenges of balancing remote work with homeschooling and childcare, coupled with increased domestic responsibilities, likely contributed to these heightened health risks, particularly for mothers.

It is noteworthy that substantial evidence indicates that women, beyond the scope of health emergencies, experience greater fear and are more likely to develop anxiety disorders than men, with socialization processes playing a moderating role in these gender differences (172). Women, often bearing the brunt of economic downturns, may experience compounded stressors that exacerbate feelings of helplessness and anxiety. This highlights the importance of considering economic factors alongside gender when addressing mental health.

Further exploring the cultural dimension, traditional gender roles have placed a disproportionate burden on women, particularly in caregiving and household responsibilities, as already mentioned. However, some studies show differences between ethnic groups suggesting that cultural context has influenced mental health outcomes. For example, in China, higher levels of risk of mental disorder were found in men than in women (92) and young married men had more psychological symptoms during the pandemic than young women (106). Taubman-Bem-Arti et al. assessed stress and anxiety associated with COVID-19 among pregnant Arab and Jewish women living in Israel and found higher rates for Arab women (88).

In addition to gender roles, it is worth mentioning the role of community and collective experiences in shaping mental health outcomes. Arab society, for example, tends to be more collective, with lower levels of education, income, and employment. Other crucial aspect is that cultural beliefs and practices play a pivotal role in shaping individuals’ understanding of mental health and illness. This is particularly relevant in contexts where western notions of psychopathology may differ from local conceptions.

Moreover, its important to mention that cultural contexts influence the stigma associated with mental illness, which can deter individuals from seeking help. For instance, women may generally be more likely to request assistance, but the ones who face stigma and prejudice, such as migrants and black women, may face systemic barriers that limit their access to mental health services. This gap between population groups warrants more substantial analyses questioning how cultural issues influence psychological status, when they are responsible for marks of inequities that are experienced in a particular way by each woman. This construction takes place in language and challenges the possibilities of being of each woman (173). In this process, women's relations with their maternal role can be deidealized, since, on the one hand, women are always in a place of failure, of not being able to, of not having control, and on the other hand, their mental health is a supposed place of sanity, health, and safety (174, 175).

However, although the present sample referred to women as a vulnerable group, it appears that most studies take a limited approach to the problem by adopting psychiatric disorders and their standardized symptoms as special parameters of analysis and preferentially focusing on the presence of depression and anxiety. Therefore, they forget to explore the meaning of the singular situations experienced by each woman.

Importantly, several variations of the expression “mental health” were used in the search strategy, and different types of study were included to cover a greater diversity of perspectives. Nonetheless, the prevalence of symptoms understood as those of mental disorders constituted the main object of analysis in the publications.

The pandemic context created limitations to the methodological processes of participant recruitment and data collection. This resulted in a scenario in which the production of knowledge resorted almost exclusively to the use of self-administered questionnaires or, more infrequently, questionnaires administered via telephone by researchers. It is worth mentioning that research on mental health often relies heavily on self-reported data, which can introduce significant methodological challenge. Participants may overreport positive attributes or underreport negative ones to present a favorable self-image. Furthermore, individuals from different socioeconomic backgrounds may have varying levels of literacy, numeracy, or cultural factors that influence their ability to understand and respond to survey questions.

Although the articles included detailed descriptions of the measurement methods, the use of scales as a key parameter for assessing participant mental health status was rarely questioned by the authors. In general, if addressed, this concern was restricted to indicating that self-administration of scales, associated with lack of validation of the results by a mental health professional, were limiting factors for assessing participant mental health.

The most privileged approaches (quantitative and based on syndromic diagnoses) allowed establishing correlations between phenomena, but they also hampered an investigation of the true network of causal relationships between them. The studies also showed an important bias in sample selection, as they were conducted basically online and included participants who might have been biased. The study samples were significantly heterogeneous in terms of population, and the outcomes and research instruments used were highly diverse, which favored part of the population with access to communication media and ability to complete self-administered scales and questionnaires.

Cross-sectional studies, performed mostly at the peak of the pandemic in different countries, provided one overview of women's mental health at this stage. However, the evaluated outcomes may be very sensitive to the health and social isolation measures applied and the evolution of the response to these measures in each country or region. It is worth noting that specific cultural issues faced by female respondents may have different impacts on their mental health.

Conversely, since methodological choices surpass the simple definition of the most adequate means to approach a given object and express the authors’ position in the face of different conceptions and understandings of reality (176), the high number of quantitative designs focused on the use of scales seems to reflect a typical approach from the field of psychopathology. It sees the phenomenon of psychological distress not as a condition experienced by human subjects in their singularities, but as manifestations of the pathological condition that cause disorders to be corrected.

Regardless of country or culture of origin, as well as marital status and age, studies revealed that women (pregnant or not) showed more exuberant psychological symptoms in the COVID-19 pandemic context, including distress, when compared to men. There are, therefore, gender differences in psychological manifestations in response to the pandemic.

However, when discussing the results, the studies established little or no interaction with theoretical references that would help expand the understanding of how psychological distress has been experienced by women throughout the pandemic. The same observation applies to the proposed strategies and interventions.

There is a prevailing interest in pregnant and postpartum women, who are the object of 43% of the studies, including three literature reviews assessing the prevalence of psychiatric symptoms in the perinatal period (77, 94, 149). However, an analysis of the study discussions shows that choosing pregnant and postpartum women as the main subjects of interest denotes a concern with the consequences of the pandemic on the development of babies and their future psychic life, with less attention to the consequences on the lives of these women. This view of sexual and reproductive health negatively affects the health care services provided to pregnant women (10).

Importantly, women's health in the 19th and 20th centuries was intended to seek, with the development of healthy bodies destined for reproduction, fetal health. Löwy describes how, since the late 19th century, medicine has sought to foster the birth of healthy children by increasing surveillance of the bodies of pregnant women (177). One of the expressions of this surveillance was named prenatal care, which grew significantly in the mid-20th century. This parameter strongly inspired feminist criticisms in the 1970s and will expand on the submission of female bodies to normative practices that are often coercive (178).

In contemporary times, prenatal care incorporates, on the one hand, the universally accepted diagnostic tests that monitor mother-fetus health and, on the other hand, more controversial tests that make fetal anomalies visible. In any case, the bodies of pregnant women would essentially be used to monitor the fetus. Similarly, this study identified a concern with the construction of maternal mental health that seems to be mostly intended to ensure the birth of psychically healthy babies, which, in theory, would guarantee a future full of mentally healthy adults over time. In our perspective, women's mental health should be regarded as a primary focus *per se*, and studies should seek to show the penalties and emotional costs of their insertion in the social bond considering their specific perspectives in conversation with other social determinants of health, such as poverty, race/ethnicity, age, and sexual orientation.

Regarding policy implications, the studies present proposals across several areas of, but most of them focused on health care, with special attention to regular communication, screening for more severe cases, and specific psychotherapies. However, what is missing in the suggestions is the development of approaches that take the particularities of women's distress into account. Sociocultural elements should be considered, such as the prominence of women in caregiving roles, and the physical, psychological, financial, and caregiving impacts on the female population. Beyond immediate healthcare, social protection measures should be guaranteed immediately, supporting the specific needs that a health emergency requiring social isolation can generate. Additionally, specific training focused on women's mental health care should be developed at national and local levels, involving professionals from different areas, with a special focus on primary health care. A critical area for future research is the role of digital mental health resources during the pandemic. Telehealth has presented opportunities and challenges for mental health service delivery, especially for underserved populations. Research should evaluate the effectiveness of these digital interventions and identify barriers to access, ensuring that all minority groups can benefit from such services.

It´s important to remember that WHO has highlighted the relevance of social determinants of health, including gender, in specific processes of consultation and agreement with counterparts, the governments of the countries, and in articulation with partners such as academia, nongovernmental organizations, and companies, among others, as previously mentioned. However, in the specific case of COVID-19, communities and health systems seem to have generally preferred to consider women as carriers of excessive signs of psychological distress than to understand such signs as important effects in response to a very serious pandemic setting in which, as stated by Birman, women's feeling of helplessness was determined by real external, family, and institutional threats (31).

The results of this study show that the place where each woman is culturally, socially, and economically situated matters in the development of a higher or lower degree of psychological distress. Even in case of previous psychiatric symptoms, women seem to reveal through their psychological and social manifestations the difficulties of living and surviving in a pandemic world that threatens their own existence and that of their loved ones. Lower income and education, limited access to goods and services, and living alone or with children were all aspects that seem to push women towards greater psychological distress, as previously reported.

What cannot be deduced from the studies analyzed is how each woman psychically organizes her singular subjectivity, who listens to her suffering, where, and how she works out her emotional issues. These are probably important questions for future research. Therefore, perhaps depression and anxiety, in processes of the magnitude of the pandemic, as described by Dunker, could be understood not as a single, extremely severe illness, but as conditions that are variable and that function as an expression of collective systemic symptoms and indicators of social distress (32).

A relevant point when addressing the field of demands of the subject and subjectivities is that overcoming difficulties related to infrastructure or the search for equality will not definitively solve the problem of women's distress, who should be able to voice their uncertainties and be heard in their loneliness due to the pandemic isolation. The association of their mental state with other social determinants of health should also be explored.

In terms of limitations, we would like to note that our analysis excluded studies involving adolescents, as discussed previously. Nonetheless, it is known that children and adolescents has been significantly impacted by the pandemic, with female adolescents exhibiting a higher risk of depression and anxiety compared to their male counterparts (179, 180). The pandemic's limitations on social interaction have had a greater negative impact on adolescents and youth, who often depend on community support for validation and belonging. Adolescents may have experienced disappointments and frustrations, both in the school and family environments. In these cases, isolation and technology formed a closed system with very little exchange in situations of affective and sexual interactions. A topic for future research could be to investigate what new mechanisms could be in place for female adolescents in the post-pandemic time.

Likewise, it is important to note the limitation of the studies with respect to minority women. Even though this group was not well represented in the studies examined in our review, literature shows that situations of stigma, prejudice, limited access to services, increased isolation, and work restrictions have disproportionately affected refugee, migrant and other racialized women, particularly those living in poverty or facing economic hardship due to COVID-19 (181–183).

## Conclusions

The post-pandemic period is an opportunity to build more integrated mental health strategies capable of addressing future public health crises. By synthesizing key findings, this study shed light on the nuanced ways in which the pandemic has affected women's mental health, contributing to the development of informed strategies and interventions to support women's mental well-being in times of adversity.

As captured by the reviewed studies, the amount of distress expressed by women, basically as anxiety and depression, seems to be more significant than that of men. However, instead of being understood as greater empathy with the experienced settings requiring immediate responses, this is considered a more vulnerable feature. Women are therefore seen as a group to be strengthened, and not as a product of relations between themselves and their symptoms, desires, and languages, or as an expression of a symbolic way of functioning in the social bond. In an attempt to protect themselves and the people around them, often in a context of inequality and greater distress in their territories of origin, they have developed actions of attention, concern, and care.

As a first conclusion, the strong and immediate reaction of women, as a group, shows that, in addition to individual responses, they had a particular position implying a deep subjective responsibility, as gender, social psychology, and psychoanalysis literatures suggest (184–187). Subjective responsibility, ie, responsibility of the subject (in this case, women), implies attesting to a deep commitment to one's desire or intention (in this case, maintenance and care of biological and social life). Women, as the study shows, adopt a position of great concern with the matters concerning their lives and those of their children and families. They are concerned about how to reconcile working and caring for themselves and their surroundings, and about how to develop actions associated with physical, psychological, and social maintenance around their own lives and those of their children, families, and community, while strongly resenting the isolation requirements. Thus, instead of reading women as “more fragile,” a more appropriate approach would be to understand this group as singularly resilient.

A second aspect to consider is that the studies on pregnant women generally aimed to find ways of diagnosing their condition and “psychologically stabilizing” them, since the disorders are believed to cause harm in the short, medium, and long terms to women, but especially to the neurobehavioral development of their fetuses. Stress as a factor that reduces women's immune capacity is also highlighted, but always referring to a biological aspect, namely the long-lasting action of stress, which is a quite limiting approach. Modulating this argumentative mode could be relevant because perinatal mental health also reveals, as previously mentioned, discourses associated with the sociocultural systems into which they are inserted.

Although studies added some complexity by including different population groups, the academic contribution to the construction of explanatory hypotheses that seek to understand and directly alleviate the distress experienced by women has been limited. Thus, from our perspective, one of the main aspects in the process of expanding the understanding of how women operate in the field of mental health would be to remove them from a place of vulnerability to introduce them in spaces that value their psychological singularities, embedded in their own cultures, where they can comprehend gender, race, age, and economic inequalities and respond to them through language in specific ways.

A third observation is that the anxieties identified in the reviewed studies do not allow us to determine any special biological condition that could justify an experience of greater distress for women. On the contrary, psychological distress generally refers to recurrent psychological manifestations that reinforce the feeling of emotional helplessness regardless of the territories and processes in which they develop. This is related to how women's singularities are structured. Thus, women should be able to access health care or other services that are prepared to listen to what each one of them has to say.

Public policies for women's mental health must be reviewed in the short, medium, and long terms and analyzed through interdisciplinary and intersectoral frameworks. Recognizing the problem, listening to it, and assessing its magnitude require, in addition to a concern with disease burden and health system costs, a comprehensive prevention and care approach that goes beyond health services and reaches other areas, such as labor. Adopting policies that focus on distress experienced by women may favor a psychological reorganization of each subject, providing them with greater autonomy and singularity when facing critical situations. In the same direction, health decision-makers need to allocate increased resources to all fields to enable specific listening and care processes for women in different territories, avoiding pathologization and considering social markers translated into the language of each one. Women need to be heard and search for their own meanings.

## Data Availability

The original contributions presented in the study are included in the article/Supplementary Material, further inquiries can be directed to the corresponding author.
