# Supplementary material for: Women's mental health and COVID-19: increased vulnerability and inequalities
Source: Front Glob Womens Health. 2024 Oct 2;5:1414355. doi: 10.3389/fgwh.2024.1414355 (PMC11480059; doi:10.3389/fgwh.2024.1414355)
Supplement: Supplementary file 1 [file Table1.docx]

**SUPPLEMENTARY MATERIALS**

**Supplementary material 1**. MeSH concepts and terms

| Concept | DeCS English |
| --- | --- |
| COVID-19 | COVID-19 |
|  | Coronavirus Infections |
| SARS-CoV-2 | SARS-CoV-2 |

| Concept | DeCS English |
| --- | --- |
| Anguish | Psychological Distress |
| Anxiety | Anxiety  Anxiety Disorders |
| Bereavement | Bereavement |
| Burnout | Burnout, Professional |
| Death | Death |
| Depression | Depression  Depression, Postpartum  Depressive Disorder  Depressive Disorder, Major |
| Fear | Fear |
| Melancholy | Depressive Disorder |
| Mental Health | Mental Health |
| Post-Traumatic Stress Disorder | Stress Disorders, Post-Traumatic |
| Psychological Stress | Stress, Psychological |
| Psychological Trauma | Psychological Trauma |
| Social Isolation | Social Isolation |
| Trauma | Trauma and Stressor Related Disorders |
| Unemployment | Unemployment |

| Concept | DeCS English |
| --- | --- |
| Violence | Violence |
|  | Violence Against Women |
|  | Gender-Based Violence |
|  | Domestic Violence |
|  | Intimate Partner Violence |

| Concept | DeCS English |
| --- | --- |
| Abortion | Abortion, Spontaneous |
|  | Abortion, Induced |
|  | Abortion, Legal |
|  | Abortion, Threatened |
| Cesarean Section | Cesarean Section |
| Childbirth | Parturition  Natural Childbirth  Delivery, Obstetric |
| Contraception | Contraception |
| Infertility | Infertility |
| Pregnancy | Pregnancy  Pregnancy Outcome |
| Puerperium | Postpartum Period |

| Study types |
| --- |
| Journal article |
| Dataset |
| Editorial |
| Clinical trial |
| Controlled clinical trial |
| Randomized controlled trial |
| Pragmatic clinical trial |
| Comparative study |
| Multicenter study |
| Observational study |
| Guideline |
| Practice guideline |
| Meta-analysis |
| Personal narrative |
| Case report |
| Technical report |
| Review |
| Systematic review |

| Languages |
| --- |
| Spanish |
| English |
| Portuguese |

| Age groups |
| --- |
| Adolescents 13-18 years |
| Adults ≥ 19 years |

**Supplementary material 2**. Search syntax

**PubMed syntax**

"(((""Coronavirus Infections""[Mesh] OR ""COVID-19""[Mesh]) OR ""SARS-CoV-2""[Mesh]) AND (((((((((((((((((""Psychological Distress""[Mesh]) OR ""Anxiety""[Mesh]) OR ""Anxiety Disorders""[Mesh]) OR ""Burnout, Professional""[Mesh]) OR ( ""Depression""[Mesh] OR ""Depressive Disorder""[Mesh] OR ""Depression, Postpartum""[Mesh] OR ""Depressive Disorder, Major""[Mesh] )) OR ""Unemployment""[Mesh]) OR ""Stress, Psychological""[Mesh]) OR ""Social Isolation""[Mesh]) OR ""Bereavement""[Mesh]) OR ""Fear""[Mesh]) OR ""Death""[Mesh]) OR ""Mental Health""[Mesh]) OR ""Trauma and Stressor Related Disorders""[Mesh]) OR ""Stress Disorders, Post-Traumatic""[Mesh]) OR (""Violence""[Mesh] OR ""Domestic Violence""[Mesh] OR ""Gender-Based Violence""[Mesh] OR ""Intimate Partner Violence""[Mesh])) OR (""Abortion, Induced""[Mesh] OR ""Abortion, Threatened""[Mesh] OR ""Abortion, Legal""[Mesh] OR ""Abortion, Spontaneous""[Mesh])) OR (((((((""Contraception""[Mesh]) OR ""Cesarean Section""[Mesh]) OR ""Infertility""[Mesh]) OR ""Parturition""[Mesh]) OR ""Natural Childbirth""[Mesh]) OR ""Delivery, Obstetric""[Mesh]) OR ""Postpartum Period""[Mesh])) AND ((casereports[Filter] OR clinicaltrial[Filter] OR comparativestudy[Filter] OR controlledclinicaltrial[Filter] OR dataset[Filter] OR editorial[Filter] OR guideline[Filter] OR meta-analysis[Filter] OR multicenterstudy[Filter] OR observationalstudy[Filter] OR personalnarrative[Filter] OR practiceguideline[Filter] OR pragmaticclinicaltrial[Filter] OR preprint[Filter] OR randomizedcontrolledtrial[Filter] OR review[Filter] OR systematicreview[Filter] OR technicalreport[Filter]) AND (humans[Filter]) AND (2020/1/1:2021/6/30[pdat]) AND (english[Filter] OR portuguese[Filter] OR spanish[Filter]) AND (adolescent[Filter] OR alladult[Filter]))) AND (""Women""[Mesh])",Most Recent,,"(""Coronavirus Infections""[MeSH Terms] OR ""COVID-19""[MeSH Terms] OR ""SARS-CoV-2""[MeSH Terms]) AND (""Psychological Distress""[MeSH Terms] OR ""Anxiety""[MeSH Terms] OR ""Anxiety Disorders""[MeSH Terms] OR ""burnout, professional""[MeSH Terms] OR (""Depression""[MeSH Terms] OR ""Depressive Disorder""[MeSH Terms] OR ""depression, postpartum""[MeSH Terms] OR ""depressive disorder, major""[MeSH Terms]) OR ""Unemployment""[MeSH Terms] OR ""stress, psychological""[MeSH Terms] OR ""Social Isolation""[MeSH Terms] OR ""Bereavement""[MeSH Terms] OR ""Fear""[MeSH Terms] OR ""Death""[MeSH Terms] OR ""Mental Health""[MeSH Terms] OR ""Trauma and Stressor Related Disorders""[MeSH Terms] OR ""stress disorders, post traumatic""[MeSH Terms] OR (""Violence""[MeSH Terms] OR ""Domestic Violence""[MeSH Terms] OR ""Gender-Based Violence""[MeSH Terms] OR ""Intimate Partner Violence""[MeSH Terms]) OR (""abortion, induced""[MeSH Terms] OR ""abortion, threatened""[MeSH Terms] OR ""abortion, legal""[MeSH Terms] OR ""abortion, spontaneous""[MeSH Terms]) OR (""Contraception""[MeSH Terms] OR ""Cesarean Section""[MeSH Terms] OR ""Infertility""[MeSH Terms] OR ""Parturition""[MeSH Terms] OR ""Natural Childbirth""[MeSH Terms] OR ""delivery, obstetric""[MeSH Terms] OR ""Postpartum Period""[MeSH Terms])) AND ((""case reports""[Publication Type] OR ""clinical trial""[Publication Type] OR ""comparative study""[Publication Type] OR ""controlled clinical trial""[Publication Type] OR ""dataset""[Publication Type] OR ""editorial""[Publication Type] OR ""guideline""[Publication Type] OR ""meta analysis""[Publication Type] OR ""multicenter study""[Publication Type] OR ""observational study""[Publication Type] OR ""personal narrative""[Publication Type] OR ""practice guideline""[Publication Type] OR ""pragmatic clinical trial""[Publication Type] OR ""preprint""[Publication Type] OR ""randomized controlled trial""[Publication Type] OR ""review""[Publication Type] OR ""systematic review""[Filter] OR ""technical report""[Publication Type]) AND ""humans""[MeSH Terms] AND 2020/01/01:2021/06/30[Date - Publication] AND (""english""[Language] OR ""portuguese""[Language] OR ""spanish""[Language]) AND (""adolescent""[MeSH Terms] OR ""adult""[MeSH Terms])) AND ""Women""[MeSH Terms]",22,16:57:43

**SCOPUS syntax**

( ( KEY ( psychological  AND distress )  OR  KEY ( anxiety )  OR  KEY ( anxiety  AND disorders )  OR  KEY ( burnout,  AND professional )  OR  KEY ( depression )  OR  KEY ( depression,  AND post  AND partum )  OR  KEY ( depressive  AND disorder )  OR  KEY ( depressive  AND disorder,  AND major )  OR  KEY ( unemployment )  OR  KEY ( stress,  AND psychological )  OR  KEY ( social  AND isolation )  OR  KEY ( bereavement )  OR  KEY ( fear )  OR  KEY ( death )  OR  KEY ( mental  AND health )  OR  KEY ( trauma  AND  stressor  AND related  AND disorders )  AND  KEY ( stress  AND disorders,  AND post-traumatic )  OR  KEY ( psychological  AND trauma )  OR  KEY ( psychological  AND distress ) ) )  AND  ( woman  OR  women )  AND  ( LIMIT-TO ( DOCTYPE ,  "ar" )  OR  LIMIT-TO ( DOCTYPE ,  "re" )  OR  LIMIT-TO ( DOCTYPE ,  "le" )  OR  LIMIT-TO ( DOCTYPE ,  "no" )  OR  LIMIT-TO ( DOCTYPE ,  "cp" )  OR  LIMIT-TO ( DOCTYPE ,  "ed" )  OR  LIMIT-TO ( DOCTYPE ,  "ch" )  OR  LIMIT-TO ( DOCTYPE ,  "sh" )  OR  LIMIT-TO ( DOCTYPE ,  "er" )  OR  LIMIT-TO ( DOCTYPE ,  "bk" )  OR  LIMIT-TO ( DOCTYPE ,  "dp" )  OR  LIMIT-TO ( DOCTYPE ,  "tb" )  OR  LIMIT-TO ( DOCTYPE ,  "Undefined" ) )  AND  ( LIMIT-TO ( PUBYEAR ,  2021 )  OR  LIMIT-TO ( PUBYEAR ,  2020 )  OR  LIMIT-TO ( PUBYEAR ,  2019 ) )  AND  ( LIMIT-TO ( EXACTKEYWORD ,  "Human" )  OR  LIMIT-TO ( EXACTKEYWORD ,  "Humans" )  OR  LIMIT-TO ( EXACTKEYWORD ,  "Female" )  OR  LIMIT-TO ( EXACTKEYWORD ,  "Adult" )  OR  LIMIT-TO ( EXACTKEYWORD ,  "Psychology" )  OR  LIMIT-TO ( EXACTKEYWORD ,  "Distress Syndrome" )  OR  LIMIT-TO ( EXACTKEYWORD ,  "Posttraumatic Stress Disorder" )  OR  LIMIT-TO ( EXACTKEYWORD ,  "Depression" )  OR  LIMIT-TO ( EXACTKEYWORD ,  "Psychological Distress" )  OR  LIMIT-TO ( EXACTKEYWORD ,  "Stress Disorders, Post-Traumatic" )  OR  LIMIT-TO ( EXACTKEYWORD ,  "Anxiety" )  OR  LIMIT-TO ( EXACTKEYWORD ,  "Mental Stress" )  OR  LIMIT-TO ( EXACTKEYWORD ,  "Mental Health" )  OR  LIMIT-TO ( EXACTKEYWORD ,  "Young Adult" )  OR  LIMIT-TO ( EXACTKEYWORD ,  "Stress, Psychological" )  OR  LIMIT-TO ( EXACTKEYWORD ,  "Adolescent" )  OR  LIMIT-TO ( EXACTKEYWORD ,  "Aged" )  OR  LIMIT-TO ( EXACTKEYWORD ,  "Cross-sectional Study" )  OR  LIMIT-TO ( EXACTKEYWORD ,  "Cross-Sectional Studies" )  OR  LIMIT-TO ( EXACTKEYWORD ,  "Anxiety Disorder" )  OR  LIMIT-TO ( EXACTKEYWORD ,  "Psychological Well-being" )  OR  LIMIT-TO ( EXACTKEYWORD ,  "Follow Up" )  OR  LIMIT-TO ( EXACTKEYWORD ,  "Self Report" )  OR  LIMIT-TO ( EXACTKEYWORD ,  "Psychotrauma" )  OR  LIMIT-TO ( EXACTKEYWORD ,  "Post-traumatic Stress Disorder" )  OR  LIMIT-TO ( EXACTKEYWORD ,  "Stress" )  OR  LIMIT-TO ( EXACTKEYWORD ,  "Fear" )  OR  LIMIT-TO ( EXACTKEYWORD ,  "Sex Difference" )  OR  LIMIT-TO ( EXACTKEYWORD ,  "Aged, 80 And Over" )  OR  LIMIT-TO ( EXACTKEYWORD ,  "Mental Disorders" )  OR  LIMIT-TO ( EXACTKEYWORD ,  "Systematic Review" )  OR  LIMIT-TO ( EXACTKEYWORD ,  "Major Depression" )  OR  LIMIT-TO ( EXACTKEYWORD ,  "Comparative Study" )  OR  LIMIT-TO ( EXACTKEYWORD ,  "Prospective Studies" )  OR  LIMIT-TO ( EXACTKEYWORD ,  "Anxiety Disorders" )  OR  LIMIT-TO ( EXACTKEYWORD ,  "Longitudinal Studies" )  OR  LIMIT-TO ( EXACTKEYWORD ,  "Emotional Stress" )  OR  LIMIT-TO ( EXACTKEYWORD ,  "Distress" )  OR  LIMIT-TO ( EXACTKEYWORD ,  "Resilience, Psychological" )  OR  LIMIT-TO ( EXACTKEYWORD ,  "Gender" )  OR  LIMIT-TO ( EXACTKEYWORD ,  "Clinical Trial" )  OR  LIMIT-TO ( EXACTKEYWORD ,  "Pain" )  OR  LIMIT-TO ( EXACTKEYWORD ,  "Resilience" )  OR  LIMIT-TO ( EXACTKEYWORD ,  "Burnout" )  OR  LIMIT-TO ( EXACTKEYWORD ,  "Depressive Disorder" ) )  AND  ( LIMIT-TO ( EXACTKEYWORD ,  "COVID-19" )  OR  LIMIT-TO ( EXACTKEYWORD ,  "Coronavirus Infection" )  OR  LIMIT-TO ( EXACTKEYWORD ,  "Coronavirus Infections" )  OR  LIMIT-TO ( EXACTKEYWORD ,  "SARS-CoV-2" ) )  AND  ( LIMIT-TO ( LANGUAGE ,  "English" )  OR  LIMIT-TO ( LANGUAGE ,  "Spanish" )  OR  LIMIT-TO ( LANGUAGE ,  "Portuguese" ) ) View less

**Supplementary material 3**. Selection criteria

| **Inclusion criteria** | **Exclusion criteria** |
| --- | --- |
| Women in general | No specific information on women |
| Health professionals | Groups of patients with specific diseases |
| Pregnant women | Groups dedicated to specific activities: athletes, teachers |
| Postpartum women |  |
| Published in English, Portuguese, or Spanish |  |
| Published in refereed journals |  |
| Published between January 1, 2020 and June 30, 2021 |  |
| Selected by 3 researchers |  |

**Supplementary material 4**. Factors associated with psychological distress

| **Factor categorization** | **Risk factor** | **Outcome** | **Protective factor** | **Outcome** |
| --- | --- | --- | --- | --- |
| Sociodemographic characteristics | Being younger (18-39 years) | Anxiety, depression, and psychological distress | More advanced age | Stress |
|  | Being over 50 years of age | Anxiety and psychological distress |  |  |
|  | Having a higher level of education | Depression |  |  |
|  | Being White | Anxiety |  |  |
|  | Being a student | Anxiety and depression |  |  |
|  | Being Brown or Black | Depression and stress |  |  |
|  | Having a lower level of education | Anxiety, depression, and psychological distress |  |  |
|  | Living in urban areas | Anxiety and depression |  |  |
| **Factor categorization** | **Risk factor** | **Outcome** | **Protective factor** | **Outcome** |
| Pandemic context | Believing you have COVID-19 (even without a diagnosis) | Depression | Adopting COVID-19 preventive measures | Depression |
|  | Having COVID-19 symptoms | Depression | Having a perception that COVID-19 is under control | Anxiety and depression |
|  | Complying with preventive measures | Anxiety | Seeking information about COVID-19 | Depression |
|  | Watching news and stories about COVID-19 on social media and television | Psychological distress | Having access to outdoor spaces | Stress |
|  | Linking COVID-19 to fetal anomalies and intrauterine fetal death | Anxiety |  |  |
|  | Having limited knowledge about COVID-19 | Psychological distress |  |  |
|  | Personally meeting someone who has COVID-19 | Anxiety and depression |  |  |
|  | Thinking someone in the family might have COVID-19 | Depression |  |  |
|  | Perceiving heightened levels of personal threat to your own health and the well-being of family members | Depression |  |  |
|  | Living in a place with a high COVID-19 mortality rate | Anxiety and depression |  |  |
|  | Having a perception that COVID-19 represents long-term physical harm | Anxiety and depression |  |  |
|  | Having concerns about not receiving necessary prenatal care | Anxiety and depression |  |  |
|  | Having concerns about the risk of infection | Anxiety, stress, and psychological distress |  |  |
|  | Having symptoms consistent with COVID-19 | Psychological distress |  |  |
|  | Having no confidence in epidemic control | Psychological distress |  |  |
|  | Longer quarantine duration | Stress |  |  |
|  | Changes in prenatal consultations | Stress |  |  |
|  | Decreased perceived social support | Anxiety and depression |  |  |
|  | Income/job loss | Anxiety, depression, and stress |  |  |
|  | Being financially insecure | Depression and psychological distress |  |  |
|  | Reduced social activity | Stress |  |  |
|  | Shortage of basic resources | Depression |  |  |
|  | Facing new health care expenses | Depression |  |  |
|  | Being in social isolation or quarantine | Anxiety and depression |  |  |
|  | Being concerned about not being prepared for childbirth due to the pandemic | Depression |  |  |
|  | Food insecurity | Depression and stress |  |  |
|  | Having no more than 1–6 rooms in the household | Anxiety and depression |  |  |
|  | Loss of routine | Stress |  |  |
|  | Loss of childcare or caring for children at home | Anxiety, depression, and stress |  |  |
|  | Having difficulty reconciling child remote schooling with work responsibilities and income | Anxiety |  |  |
|  | Reduced access to basic goods and services | Stress and psychological distress |  |  |
|  | Strained relationship associated with social isolation | Depression |  |  |
|  | Having increased medication use | Anxiety and depression |  |  |
|  | Having reduced physical and leisure activities | Anxiety and depression |  |  |
|  | Having had a higher negative impact on quality of life | Depression |  |  |
|  | Discontinued fertility treatment | Depression |  |  |
|  | Having experienced domestic violence during the pandemic | Depression |  |  |
|  | Using social media more heavily | Depression |  |  |
|  | Reduced or no internet usage | Depression |  |  |
|  | Living in inappropriate spaces | Anxiety, depression, and psychological distress |  |  |
| **Factor categorization** | **Risk factor** | **Outcome** | **Protective factor** | **Outcome** |
| Perceptions and attitudes | Difficulty concentrating | Depression | Meditating | Anxiety and depression |
|  | Being less resilient | Psychological distress | Keeping a routine | Depression |
|  | Mood swings | Depression | Exercising | Depression |
|  | Feeling of guilt | Depression | Higher self-control | Psychological distress |
|  | Feelings of loneliness, irritation, or anger | Depression | Engaging in healthy behaviors | Stress |
|  | Having a dismissive and fearful avoidant attachment style | Postpartum depression | Having a higher level of resilience | Postpartum depression |
|  | Using emotion- and problem-focused coping strategies | Psychological distress |  |  |
| **Factor categorization** | **Risk factor** | **Outcome** | **Protective factor** | **Outcome** |
| Social relationships | Unusual family environment | Depression | Having a partner | Anxiety and depression |
|  | Family conflicts | Anxiety, depression, and stress | Having a partner who offers support | Depression |
|  | Having difficulty reconciling child remote schooling with work responsibilities and income | Depression | Family cohesion | Anxiety, depression, and postpartum depression |
|  | Being in an informal relationship | Anxiety and psychological distress | Social support | Anxiety, depression, and psychological distress |
|  | Larger family | Stress | Having children | Anxiety and depression |
|  | Spending long hours on housework and care | Psychological distress | Perception of social support | Psychological distress |
|  | Living with children | Anxiety and depression |  |  |
|  | Living abroad | Depression |  |  |
|  | Living alone | Anxiety and depression |  |  |
|  | Making video calls with family members | Psychological distress |  |  |
|  | Caring for children | Depression |  |  |
|  | Being single | Anxiety, depression, and psychological distress |  |  |
|  | Having fewer children | Depression |  |  |
|  | Strained marital relationship | Anxiety |  |  |
| **Factor categorization** | **Risk factor** | **Outcome** | **Protective factor** | **Outcome** |
| Health | Persistent burnout | Anxiety | Advanced gestational age | Anxiety and depression |
|  | Receiving psychological help | Psychological distress | Having a positive pregnancy experience | Depression |
|  | Panic attacks | Depression | Having healthy behaviors | Depression |
|  | Increased pregnancy-related stress | Depression | Childbirth during strict isolation | Postpartum depression |
|  | Low level of physical activity | Psychological distress | Having health team support throughout pregnancy and the postpartum period | Postpartum depression |
|  | Preexisting mental health problems | Anxiety, depression, and psychological distress | Silence in the postnatal ward | Postpartum depression |
|  | Disagreement with discontinuation of fertility treatment | Psychological distress | Having experienced subjective well-being prior to the pandemic | Depression |
|  | Sleep disorders | Depression and psychological distress |  |  |
|  | Chronic disease | Anxiety, stress, and depression |  |  |
|  | Perceived pain during childbirth | Postpartum depression |  |  |
|  | Pregnant or postpartum woman | Anxiety and depression |  |  |
|  | Being in the first pregnancy trimester | Anxiety and psychological distress |  |  |
|  | Perceived general stress | Depression and psychological distress |  |  |
|  | Complications in current pregnancy | Depression, stress, and psychological distress |  |  |
|  | Being underweight before pregnancy | Anxiety and depression |  |  |
|  | Pregnant women aged < 35 years | Depression |  |  |
|  | Primary pregnant women | Anxiety and depression |  |  |
|  | Pregnancy-specific stress level | Psychological distress |  |  |
|  | Pregnant women with a previous psychiatric diagnosis | Depression |  |  |
|  | History of self-reported anxiety and/or depression | Post-traumatic stress |  |  |
|  | Early gestational age | Depression |  |  |
|  | Childbirth during the pandemic | Postpartum depression |  |  |
|  | Suicidal thoughts | Depression |  |  |
|  | Sexual problems | Depression |  |  |
|  | Feeling of helplessness following discontinuation of fertility treatment | Psychological distress |  |  |
|  | Psychosomatic symptoms | Depression |  |  |
|  | Experiencing pregnancy-specific stress | Anxiety |  |  |
|  | Having a history of sexual abuse | Stress |  |  |
|  | Having a history of anxiety and depression | Anxiety and depression |  |  |
| **Factor categorization** | **Risk factor** | **Outcome** | **Protective factor** | **Outcome** |
| Work and income | Living on a low income | Anxiety, depression, and psychological distress | Having a lower socioeconomic status | Depression |
|  | Being unemployed | Anxiety and depression |  |  |
|  | Working outside the home | Psychological distress |  |  |
